# Comparative Gut Microbiome Differences between Ferric Citrate and Calcium Carbonate Phosphate Binders in Patients with End-Stage Kidney Disease

**DOI:** 10.3390/microorganisms8122040

**Published:** 2020-12-20

**Authors:** Ping-Hsun Wu, Po-Yu Liu, Yi-Wen Chiu, Wei-Chun Hung, Yi-Ting Lin, Ting-Yun Lin, Szu-Chun Hung, Rachel Ann Delicano, Mei-Chuan Kuo, Chun-Ying Wu

**Affiliations:** 1 Division of Nephrology, Department of Internal Medicine, Kaohsiung Medical University Hospital, Kaohsiung Medical University, Kaohsiung 807, Taiwan; 970392@kmuh.org.tw (P.-H.W.); chiuyiwen@kmu.edu.tw (Y.-W.C.); mechku@kmu.edu.tw (M.-C.K.); 2 Graduate Institute of Clinical Medicine, College of Medicine, Kaohsiung Medical University, Kaohsiung 807, Taiwan; 3 Faculty of Medicine, College of Medicine, Kaohsiung Medical University, Kaohsiung 807, Taiwan; 4 Department of Internal Medicine, National Taiwan University College of Medicine, Taipei 106, Taiwan; poyu.liu@gmail.com; 5 Faculty of Renal Care, College of Medicine, Kaohsiung Medical University, Kaohsiung 807, Taiwan; 6 Department of Microbiology and Immunology, Kaohsiung Medical University, Kaohsiung 807, Taiwan; wchung@kmu.edu.tw; 7 Department of Family Medicine, Kaohsiung Medical University Hospital, Kaohsiung 807, Taiwan; 8 Division of Nephrology, Taipei Tzu Chi Hospital, Buddhist Tzu Chi Medical Foundation, School of Medicine, Tzu Chi University, Hualien 970, Taiwan; water_h2o_6@hotmail.com (T.-Y.L.); szuchun.hung@gmail.com (S.-C.H.); 9 Institute of Surgical Sciences, Uppsala University, 75185 Uppsala, Sweden; rachel.delicano@surgsci.uu.se; 10 Institute of Biomedical Informatics, Medical College, National Yang-Ming University, Taipei 112, Taiwan; dr.wu.taiwan@gmail.com; 11 Department of Medical Research, Division of Translational Research, Taipei Veterans General Hospital, Taipei 112, Taiwan; 12 Department of Public Health, China Medical University, Taichung 406, Taiwan; 13 National Institute of Cancer Research, National Health Research Institutes, Miaoli 350, Taiwan

**Keywords:** microbiome, ferric citrate, calcium carbonate, phosphate binders, hemodialysis

## Abstract

Gut dysbiosis in patients with chronic kidney disease (CKD) may induce chronic inflammation and increase morbidity. Phosphate-binding agents, generally used in patients with CKD, may potentially change the composition of the gut microbiota. This study aimed to compare the microbiota composition in hemodialysis patients treated with ferric citrate or calcium carbonate. The stool microbiota was investigated in hemodialysis patients treated with ferric citrate (*n* = 8) and calcium carbonate (*n* = 46) using 16S rRNA gene amplicon sequencing profiling using linear discriminant analysis of effect size. Further predictive functional profiling of microbial communities was obtained with Tax4Fun in R. Hemodialysis patients treated with calcium carbonate had a significantly reduced microbial species diversity (Shannon index and Simpson index) and an increased microbial alteration ratio compared with patients treated with ferric citrate. A distinct microbial community structure was found in patients treated with ferric citrate, with an increased abundance of the *Bacteroidetes* phylum and a decreased abundance of the phylum *Firmicutes*. Members of the order *Lactobacillales* were enriched in patients treated with calcium carbonate, whereas taxa of the genera *Ruminococcaceae UCG-004*, *Flavonifractor*, and *Cronobacter* were enriched in patients treated with ferric citrate phosphate binder. In conclusion, Ferric citrate therapy results in a more diverse microbiome community compared to calcium carbonate therapy in hemodialysis patients with phosphate binder treatment. The gut microbiome reflects the phosphate binder choice in hemodialysis patients, further affecting the physiological environment in the gastrointestinal tract.

## 1. Introduction

Patients with chronic kidney disease (CKD) are often treated with oral phosphate binders such as iron or calcium-containing phosphate binders to control hyperphosphatemia [1]. Oral phosphate binders were used to prevent the dietary phosphate absorption within the gastrointestinal tract by exchanging an active cation with anion phosphate to make a non-absorbable compound excreted in the feces [2]. Long-term treatment could lead to gut microbial composition changes by lowering the intestinal phosphate burden [3,4]. However, the influence of iron and calcium-containing phosphate binders on the gut microbiome is still unknown. Different groups of phosphate binders that can act differently on the gut microbiome have not been investigated.

An increased microbiome diversity was observed after ferric citrate treatment in a CKD animal model. Moreover, ferric citrate treatment increased levels of tryptophanase-possessing bacteria (*Verrucomicrobia*, *Clostridiaceae,* and *Enterobacteriaceae*) [5]. However, as the gut microbiota of rats and humans are different, the impact of ferric citrate therapy in hemodialysis (HD) patients warrants further investigation. Therefore, this study aimed to explore and compare the biodiversity and composition of the gut microbiome communities in HD patients with two different phosphate binders treatment (ferric citrate versus calcium carbonate). The study findings could potentially contribute to integrating personalized medicine in phosphate binder treatment.

## 2. Materials and Methods

### 2.1. Study Participants

From August 2016 through September 2017, 85 participants were recruited from the Kaohsiung Medical University Hospital HD unit. Eligible participants were aged between 30 and 80 years and received regular HD three times per week for more than 90 days. Each HD section was 3.5–4 h with high-flux dialyzers, with a blood flow rate between 250 and 300 mL/min, dialysate flow of 500 mL/min, and single pool K*t*/V was more than 1.2 per week. Participants with partial or total colectomy or participants who were prescribed antibiotics within three months before enrollment were not enrolled in our study. Participants who used more than one type of phosphate binder were excluded (*n* = 31). Fecal samples were collected for fecal microbiota analysis by high-throughput 16S ribosomal RNA gene sequencing of ferric citrate (*n* = 8) and calcium carbonate (*n* = 46) users. All participants were treated with the single phosphate binder (ferric citrate or calcium carbonate) for at least two months.

### 2.2. Ethical Considerations

The study protocols were approved by the Ethics Committee of Kaohsiung Medical University Hospital (KMUHIRB-E(I)-20160095 approved on 8 August 2016 and KMUHIRB-E(I)-20180118 approved on 26 April 2018), and all participants provided written informed consent.

### 2.3. Comorbidity, Laboratory and Clinical Variables

Sociodemographic data, age, sex, dialysis vintage, arteriovenous shunt type, medical history, medications, and biochemical data were obtained for all participants from electronic health care system records. The definition of hypertension was 140/90 mmHg or higher or taking antihypertensive drugs. Diabetes was defined as HbA1C 6.5% or higher or taking antidiabetics. History of dyslipidemia, coronary artery disease, and cerebrovascular disease was based on physician diagnosis. Blood samples were obtained after overnight fasting through the arteriovenous fistula or graft immediately before their scheduled HD session at a single midweek dialysis session. Biochemical data for HD patients included serum values for hemoglobin, albumin, alkaline phosphatase, ion calcium, phosphate, magnesium, parathyroid hormone, and aluminum from routine blood samples obtained less than 30 days before stool sample collection. A licensed dietitian also recorded a modified short-form food frequency dietary questionnaire.

### 2.4. Fecal Sample Collection and Bacterial 16S rRNA Amplicon Sequencing and Processing

The stool samples were frozen immediately and delivered using cooler bags within 24 h to the laboratory (Germark Biotechnology, Taichung, Taiwan). DNA was extracted using a QIAamp DNA Stool Mini Kit (Qiagen, Germantown, MD, USA) and stored at −80 °C for 16S rRNA gene sequencing (at least 500 ng per sample). The DNA quality and concentration were measured by agarose gel electrophoresis and NanoDrop ND-1000 (Thermo Fisher Scientific, Waltham, MA, USA) with standards of ≥500 ng, 260/280 ratio of 1.7–1.8, and 260/230 ratio of 1.8–2.2 before downstream processing. The extracted DNA was used as the template to amplify the variable regions 3 and 4 (V3–V4) of the 16S rRNA gene using barcode-indexed PCR primers (341F and 805R) [6]. Library construction and sequencing of amplicon DNA samples were conducted by Genomics BioScience (Taipei, Taiwan). A pair-end sequencing library (insert size of 465 bp for each sample) was constructed with the sheared fragments using the TruSeq Nano DNA Library Prep kit (Illumina, San Diego, CA, USA). The purified amplicons with different index sequences were pooled in equimolar amounts and then sequenced on the Illumina MiSeq sequencer with the MiSeq Reagent Kit v3 (Illumina, San Diego, CA, USA). To reduce batch effects, all samples were sequenced simultaneously in the same laboratory (Germark Biotechnology, Taichung, Taiwan,).

The 16S-amplicon processing pipeline was modified from 16S Bacteria/Archaea SOP v1 of Microbiome Helper workflows [7]. Paired-end reads were merged raw reads using Paired-End reAd mergeR (PEAR; version 0.9.8) [8] and filtered low-quality reads by thresholds of sequence length ≥400 bp and quality score of 90% bases of reads ≥20. Processing the raw sequencing reads was demultiplexed and quality filtered using Quantitative Insight Into Microbial Ecology (QIIME; version 1.9.1) software [9].

After filtering, the sequences with 97% similarity of operational taxonomic units (OTU) from SILVA (version 123) taxonomic database [10,11] were clustered using the UCLUST algorithm [12] with a 97% sequence identity threshold. Reads were dereplicated, and singletons were discarded. The final OTU table was rarefied into minimum sequencing depth in the dataset.

### 2.5. Statistical and Bioinformatics Analyses of Microbiota

Differences in demographic characteristics between the two phosphate binder groups were determined using Student’s *t*-test or chi-squared test, as appropriate. The α-diversity (i.e., bacterial diversity within one sample) was determined by the Shannon index and Simpson index using the R “vegan” package. The β-diversity (i.e., diversity in bacterial composition between samples) was estimated by computing the Bray-Curtis distance and was visualized through a Principal Coordinates Analysis (PCoA) to evaluate the differences and similarities of bacterial communities [13]. Sample-grouped heterogeneity of β-diversity analysis was examined using analysis of similarity (Permutational multivariate analysis of variance using distance matrices (PERMANOVA)) with 10^4^ bootstrap replications. The microbial alteration ratio modified from the microbial dysbiosis index (MDI) [14] was defined as the log_10_ of the total abundance in organisms increased in calcium carbonate users divided by the total abundance of organisms decreased in ferric citrate users.

Co-correlation analysis was used to determine the relationships within a complex community. Bacteria work as functional groups (guilds) in the gut ecosystem, which leads to a microbiota community structure that can be evaluated using the Sparse Correlations for Compositional data (SparCC) algorithm by calculating the most abundant OTUs that are shared by at least 20% of the samples. Then, a co-occurrence network is built from the SparCC calculations [15]. The *p*-values were calculated using a bootstrap procedure with 100 random permutations and iterations for each SparCC calculation, and then correlation matrices were computed from the resampled data matrices. Once the bootstrapped correlation scores were computed, only OTUs with correlation scores higher than 0.3 were classified into co-abundance groups (CAGs); these coefficients were also used to assess the length of edges on the network. The latter was conducted with the fast greedy modularity optimization algorithm to identify clusters in the network. An undirected network, weighted by SparCC correlation magnitude, was generated using the R “igraph” package. Nodes with <5 connection degrees were removed from the network, and hub nodes from each cluster were extracted for further community structure analyses [16]. The closeness and eigenvector of the nodes were calculated to measure node centralities in each network. Significant associations were defined as positive SparCC correlations with a *p*-value < 0.05.

Differential abundance analysis was performed based on the relative abundance levels of the top genera, families, and orders distinctly distributed between the two phosphate binders. We further analyzed the bacterial community difference between phosphate binder groups by the linear discriminant analysis (LDA) of effect size (LEfSe) analysis at the OTU level with more than 0.1% relative abundances and present in >30% of samples to determine the most discriminatory taxa among phosphate binder users. The LEfSe analysis employed the non-parametric factorial Kruskal-Wallis test or Wilcoxon rank-sum test and LDA to identify differentially abundant taxa between the two phosphate binder groups. Only taxa with an LDA score more significant than two or less than two at a *p* < 0.05 were considered significantly enriched. All statistical tests were two-tailed, and *p* < 0.05 was considered statistically significant. Furthermore, the heat tree method was used to compare the abundance of different taxonomic levels for each pair of factors in a metadata variable. A hierarchical structure of taxonomic classifications to quantitatively (median abundance) and statistically (non-parameter Wilcoxon rank-sum test) depict taxa differences among communities was performed using R “metacoder” package [17]. Statistical analyses were performed using R statistical software (version 3.5.1).

### 2.6. Functional Annotation

Predicted functional genes were aligned to the Kyoto Encyclopedia of Genes and Genomes (KEGG) database and annotated by KEGG orthology (KO) using the R “Tax4Fun” package [18]. The KEGG metabolic modules were retrieved from the KEGG MODULE database, mapped with KOs, and calculated the differential abundance between two phosphate binders by Wilcoxon rank-sum test. KEGG modules were deemed present when ≥30% of the enzymes were recovered after manual removing overly “promiscuous” enzymes (that is, present in multiple modules) before the abundance calculation.

In total, 85 HD patients provided a stool sample. After excluding participants who used two types of phosphate binders (*n* = 31), HD patients taking ferric citrate (*n* = 8) and calcium carbonate phosphate binders (*n*= 46) were compared, as shown in Figure S1. The baseline characteristics of the enrolled HD patients are reported in Table 1. The mean age was 55.8 years in the ferric citrate group and 61.5 years in the calcium carbonate group, with a mean HD duration of 91.5 months in ferric citrate users and 84.11 months in calcium carbonate users. The two groups were similar in age, gender, cause of end-stage renal disease, HD arteriovenous shunt type, comorbidities, medications known to affect the gut microbiota, the dietary consumption patterns of vegetables or fruits, and Bristol stool scale. In addition, there was no significant difference in hemoglobin, albumin, alkaline phosphatase ion calcium, phosphate, and parathyroid hormone level between groups, as shown in Table 1.

## 3. Results

### 3.1. Patient Characteristics

In total, 85 HD patients provided a stool sample. After excluding participants who used two types of phosphate binders (*n* = 31), HD patients taking ferric citrate (*n* = 8) and calcium carbonate phosphate binders (*n* = 46) were compared (Figure S1). The baseline characteristics of the enrolled HD patients are reported in Table 1. The mean age was 55.8 years in the ferric citrate group and 61.5 years in the calcium carbonate group, with a mean HD duration of 91.5 months in ferric citrate users and 84.11 months in calcium carbonate users. The two groups were similar in age, gender, cause of end-stage renal disease, HD arteriovenous shunt type, comorbidities, medications known to affect the gut microbiota, the dietary consumption patterns of vegetables or fruits, and Bristol stool scale. In addition, there was no significant difference in hemoglobin, albumin, alkaline phosphatase ion calcium, phosphate, and parathyroid hormone level between groups, as shown in Table 1.

### 3.2. Gut Microbiota Profile Differs between Ferric Citrate and Calcium Carbonate Treatment

**Figure 1:**
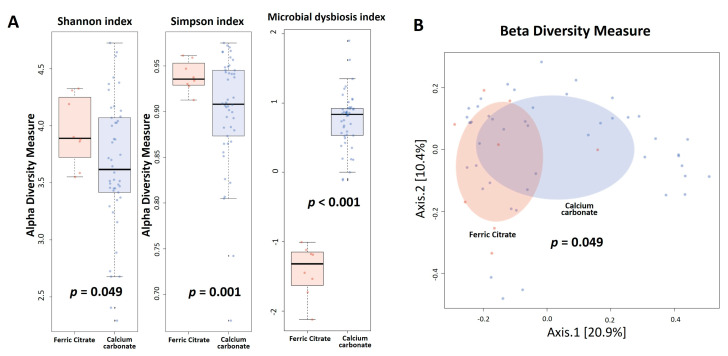
Hemodialysis patients with calcium carbonate phosphate binders had lower α diversity (Shannon index and Simpson index), higher microbial dysbiosis index (**A**), and different β diversity (Bray-Curtis distance metrics) (**B**) compared to the ferric citrate phosphate binders used.

No differences in gene rarefaction were found between two phosphate binders (Figure S2). To evaluate alterations in the microbiota structure between treatments, microbial α diversity (i.e., within-sample diversity) and β diversity (i.e., diversity between samples) were measured, with a significant decrease in the α diversity using the Shannon index (*p* = 0.049) and Simpson index (*p* = 0.001) in the calcium carbonate group (Figure 1A). The microbial alteration ratio was higher in calcium carbonate users compared to ferric citrate users (*p* < 0.001), as shown in Figure 1A. β diversity (bacterial community) was calculated using Bray-Curtis distance metrics and visualized in PCoA plots, with the microbiota composition of ferric citrate users significantly different from that of calcium carbonate users (PERMANOVA, *p* = 0.049) (Figure 1B).

### 3.3. Co-Occurrence Pattern Analysis of the Intestinal Ecosystems of Ferric Citrate Users or Calcium Carbonate Users

For visualization of the internal interactions and further measurement of the microbial community, SparCC was used to calculate the Spearman correlation coefficient with the corresponding *p*-value between every two taxa. Co-occurrence analysis was used to investigate the potential relationships between different taxa in ferric citrate and calcium carbonate users. Each node in the network indicates a bacterial genus (Figures S3 and S4, Tables S1 and S2). The complexity and composition of the networks were similar for both phosphate binder groups. The highest identifying hub species within each ecosystem showed that *Bacteroidetes* was a potential keystone phylum determining the network in both phosphate binder groups. The identified centers (hubs) of core microbes included the species belong to genera *Bacteroides* in ferric citrate users and the species belong to genera *Prevotella 9* in calcium carbonate users (Tables S1 and S2).

### 3.4. Specific Microbial Taxa are Associated with Different Phosphate Binders

**Figure 2:**
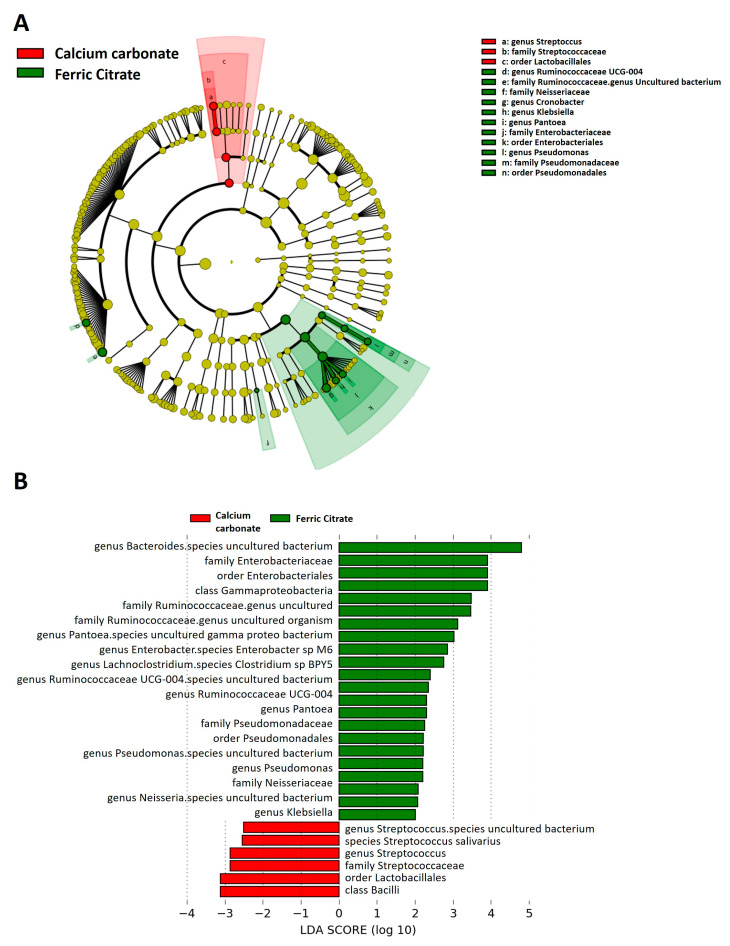
Linear discriminant analysis Effect Size (LEfSe) showing microbiome differences between ferric citrate and calcium carbonate phosphate binder users at various taxonomic levels. (**A**) Cladogram demonstrating microbiome differences at various phylogenic levels, (**B**) LEfSe analysis with linear discriminant analysis (LDA) score representing statistical and biological differences in taxa between groups. Green indicates taxa enriched in a ferric citrate group and red indicates taxa enriched in a calcium carbonate phosphate binder group.

To identify the significant differentiating taxa between study groups, we performed a discriminant analysis using LEfSe and cladogram, showing enrichment in *Gammaproteobacteria* taxa in ferric citrate users, including the genera *Pantoea*, *Enterobacter*, *Neisseria*, and *Klebsiella* (all from order *Enterobacterales*) and the genus *Pseudomonas* (family *Pseudomonadaceae*); the genera *Bacteroides* (phylum *Bacteroidetes*), *Lachnoclostridium*, *Ruminococcaceae UCG-004* (phylum *Firmicutes*) (Figure 2A,B). *Streptococcus* and *Lactobacillales*, belonging to the phylum of *Bacilli* as well as lactate producing bacteria, were enriched in the fecal microbiota of HD patients treated with calcium carbonate (Figure 2A,B). After linear discriminant analysis, the differentially enriched relative abundance demonstrated increased genus *Streptococcus*, family *Streptococcaceae*, and order *Lactobacillales* in calcium carbonate users (Figure S5). Regarding ferric citrate users, increased relative taxa abundance of family *Bacteroidaceae* was observed (Figure S5).

**Figure 3:**
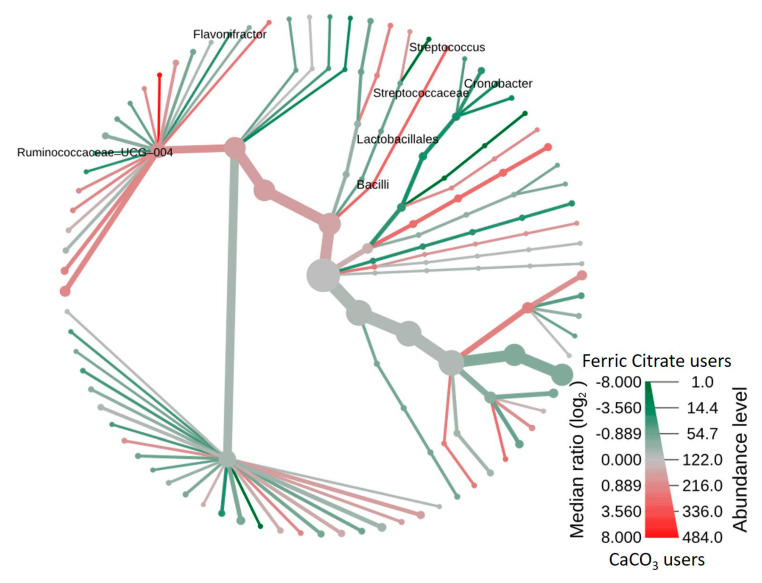
Heat tree depicting the effects of phosphate binder treatment on the intestinal microbiomes of hemodialysis patients. In comparison between ferric citrate and calcium carbonate phosphate binder, members of the class *Bacilli* were prominent in calcium-containing phosphate binder, whereas taxa of the genus *Ruminococcaceae UCG-004*, *Flavonifractor*, and *Cronobacter* were enriched in ferric citrate phosphate binder users. The heat tree was generated using Metacoder.

**Figure 4:**
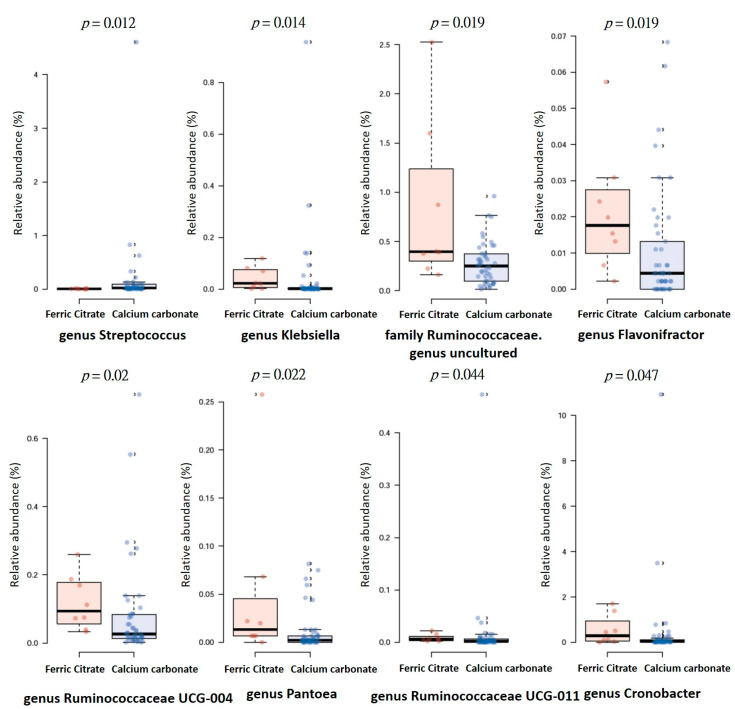
The distribution of top eight genera abundance difference between hemodialysis patients with ferric citrate or calcium carbonate phosphate binders treatment.

Furthermore, the heat tree method showed that compared to the ferric citrate users, the most abundant taxa among calcium carbonate users was class *Bacilli*, order *Lactobacillales*, family *Streptoccaceae*, genus *Streptococcus*, and species *Streptococcus salivarius* (Figure 3), whereas genus *Ruminococcaceae UCG-004*, *Flavonifractor*, and *Cronobacter* were enriched in ferric citrate phosphate binder users (Figure 3). The top eight genera abundance difference demonstrated the specific microbial features at the genus level associated with phosphate binder exposure, with significantly increased genera *Klebsiella*, *Flavonifractor*, *Pantoea*, *Cronobacter*, *Ruminococcaceae UCG-004*, *Ruminococcaceae UCG-011*, and one *Ruminococcaceae* with the unclassified genus in ferric citrate users, whereas calcium carbonate users had a higher relative abundance of genus *Streptococcus* (Figure 4).

### 3.5. Functional Characterization of the Microbiome in Different Phosphate Binder Users

To characterize the distinct functions of the gut microbiota, we performed functional annotations of the metagenome to KEGG modules. The predicted KEGG modules that were significantly enriched in calcium carbonate users related to metabolism, including pantothenate biosynthesis, C1-unit interconversion, and formaldehyde assimilation (serine pathway) (Figure S6). In contrast, the predicted KEGG modules significantly enriched in ferric citrate users included cholesterol biosynthesis, clavaminate biosynthesis, enterotoxigenic *Escherichia coli* pathogenicity signature, and vibrio cholera pathogenicity signature (Figure S6).

## 4. Discussion

In our study, the stool microbial communities in HD patients receiving ferric citrate and calcium carbonate were structurally different, with reduced microbial species diversity and increased microbial dysbiosis index in calcium carbonate users. The distinctiveness of the microbiota was confirmed by β diversity analysis (Bray-Curtis dissimilarity metrics), demonstrating the clustering of samples according to two different phosphate binders, with the microbial communities in both groups containing higher levels of *Bacteroidetes* and lower levels of *Firmicutes*, similar to the microbial communities identified in rats with CKD [5]. In terms of the stool microbiota composition, calcium carbonate users had more bacteria from order *Bacilli* to species *Streptococcus salivarius*; specifically, genus *Streptococcus* was enriched in calcium carbonate users, while the genus *Ruminococcaceae UCG-004*, *Flavonifractor*, and *Cronobacter* were enriched in ferric citrate users.

The co-occurrence analysis identifies groups of organisms that may have commensal interactions with each other or, conversely, organisms that may be antagonistic to each other in a given environmental setting. Centralized modules within a correlation network may also indicate “hub species”: organisms whose presence may be crucial to the stability of a community [16]. Here, we found hub species among two phosphate binders belong to two essential genera *Bacteroides* and *Prevotella* and may represent different enterotypes (*Bacteroides*/*Prevotella*). These hub species are vital to preserving their ecological communities’ organization and diversity through biotic interactions with other ecosystem members [19]. The identified hub species in the co-occurrence network showed the species belong to genera *Bacteroides* in ferric citrate users and the species belong to genera *Prevotella 9* in calcium carbonate users. The dominant hub species belong to genera *Bacteroides* in ferric citrate users may explain by the iron-containing environment in the gut [20]. The gut anti-acid effect by calcium carbonate may explain the dominant hub species belong to genera *Prevotella* in our study [21]. Thus, relatively small differences in individual diets or drugs could affect hub species, which may be essential to organize gut microbial consortia into distinct types of communities, or “enterotypes” [22,23]. As demonstrated in our study, the different phosphate binders treatment could present different hub species in HD patients.

Iron-based phosphate binders had been proposed to alter the gut microbiota [24]. Limited evidence demonstrated calcium-based phosphate binders’ effect on the gut microbiota in the literature [24]. In our study, *Bacteroidaceae*, a typical and large family of gastrointestinal Gram-negative bacteria, was enriched in ferric citrate users. Genus *Bacteroides* was also identified as hub taxa in the co-occurrence analysis of ferric citrate users, whereas *Streptococcaceae*, a family of Gram-positive bacteria, was predominant in calcium carbonate users. In particular, *Streptococcus salivarius*, which needs calcium carbonate as essential material [25] and can produce active urease [26], was more relatively abundant in calcium carbonate users. Importantly, *Streptococcus* is associated with hypertension [27], atherosclerotic cardiovascular diseases [28], heart failure [29], and atrial fibrillation [30], which are key comorbidities in HD patients. Furthermore, the functional prediction of the gut microbiome demonstrated an increased pathogenicity signature of enterotoxigenic *Escherichia coli* among KEGG modules.

Interestingly, citrate is a common biomolecule that chelates Fe(III) and can be used by some bacteria (e.g., *Escherichia coli* [31,32] and *Bacillus cereus* [33]) as an iron-chelating molecule to fulfill their nutritional requirement for iron. The receptors specific for the uptake of ferric citrate are typically grouped with iron-loaded siderophore receptors on the bacterial cell surface [34]. In our study, *Cronobacter*, present in the siderophore-mediated iron acquisition system [28], was enriched in ferric citrate users, confirming the link between ferric citrate exposure and Gram-negative bacteria richness.

In a recent study of 5/6 nephrectomized rats that underwent 6 weeks of a 4% ferric citrate diet, the ferric citrate diet increased fecal α-diversity (species richness), reduced the relative abundance of *Firmicutes*, and increased the relative abundance of the *Akkermansia* genus and the *Clostridiaceae* and *Enterobacteriaceae* families compared to the untreated CKD rats [5]. We also demonstrated the increased α-diversity in ferric citrate users as well as lower MDI. Comparing the specific taxa between phosphate binders, ferric citrate users had a lower relative abundance of *Firmicutes* and a higher relative abundance of *Bacteroidetes*; however, there was no difference in family *Enterobacteriaceae* Figure S5 or *Clostridiaceae* (*p* = 0.428); rather, there was an increased relative abundance of family *Bacteroidaceae*, and genus *Ruminococcaceae UCG-004*, *Flavonifractor*, and *Cronobacter* in ferric citrate users. In this study, identifying the phosphate binder effect on the gut opens a new era in nephrology, filling the existing gap in the interpretation of the beneficial effects of phosphate binders.

Oral iron supplementation can reduce beneficial bacterial such as *Bifidobacteriaceae* and *Lactobacillaceae* and increase gut permeability [35]. Contrary to our results, an in vitro study on Caco-2 cells incubated with ferric citrate/ferrous sulfate and human microbiota showed diminished levels of *Lactobacillaceae* and *Bifidobacteriaceae* [36]. In normal and CKD rats treated with ferric citrate, decreased *Lactobacillaceae* levels were observed [5]. In our study, there was no difference in the microbiome family *Bifidobacteriaceae* (*p* = 0.240) between treatments, with an extremely low abundance of *Lactobacillaceae* but a more significant difference between two phosphate binders, including genera *Ruminococcaceae UCG-004*, *Flavonifractor*, *Cronobacter*, and *Streptococcus*. Dietary iron depletion reduced *Ruminococcaceae* in a mouse model [37], and the oral administration of liquid iron increased *Ruminococcaceae* in a rat model [38], suggesting the detrimental effects of excess iron on gut health. The *Ruminococcaceae* family degrades mucus, and complex carbohydrates can produce butyrate production [22,39] and are negatively associated with pulse wave velocity [40], which is an indicator of arterial stiffness, whereas the genus *Flavonifractor* potentially induces oxidative stress and inflammation via cleavage of the flavonoid C-ring and degeneration of quercetin [41,42]. A positive correlation between *Flavonifractor* and circulating inflammatory markers were also found [43]. However, ferric citrate treatment did not promote inflammation in a randomized clinical trial of dialysis patients [44].

Evaluation of the KEGG modules functional prediction showed the enriched function of cofactor and vitamin biosynthesis in calcium carbonate users compared to ferric citrate users, such as pantothenate biosynthesis and C1-unit interconversion. Pantothenate (Pantothenic acid), a water-soluble vitamin B5, is involved in the synthesis of coenzyme-A, lipids, proteins, carbohydrates, neurotransmitters, steroid hormones, and hemoglobin [45,46]. C1-unit is produced from the degradation of amino acids, glycine, serine, histidine, and tryptophan, and is involved in folate synthesis [47]. Formaldehyde assimilation (serine cycle) is involved in methanol and carbon metabolism [48,49]. Regarding the functional prediction of ferric citrate users, cholesterol biosynthesis and clavaminate biosynthesis were enriched. Several studies have confirmed that gut microbiota plays a crucial role in cholesterol metabolism [50,51]. Clavaminate synthase contains nonheme iron, which corresponds to ferric citrate treatment in our study [52].

In this study, LEfSe analysis was performed. However, one drawback is that the discriminant analysis is quite sensitive to outliers, so we also demonstrated the selected taxa’s box plot after linear discriminant analysis (Figure S5). Interestingly, we only found genus *Streptococcus*, family *Streptococcaceae*, and order *Lactobacillales* enriched abundance in calcium carbonate users, and family *Bacteroidaceae* enriched abundance in ferric citrate users. Some limitations should be acknowledged. First, cross-sectional studies provide an overview of the relative abundance of bacterial taxa at a single time point rather than capture the complex dynamics of the microbial ecosystems in the gut of HD patients with different phosphate binders. Second, confounders cannot be excluded, and statistical correlations between phosphate binders and gut microbiota profiles do not necessarily implicate a causal inference. Phosphate control treatments are essential for HD patients in daily practice, so it was challenging to include subjects without a phosphate binder. Thus, the comparison of the gut microbiota before and after phosphate binder treatment is difficult. However, further studies to compare microbiota composition between phosphate binder naïve treatment patients are still needed to elucidate the causal relationship.

Furthermore, the dietary information obtained from the questionnaire is subjective to recall bias. Our diet questionnaire was also limited to record the citrate intake among participants. The precision of dietary intake can benefit from having more objective dietary records. Third, other phosphate binders (e.g., lanthanum carbonate, sevelamer hydrochloride/carbonate, or sucroferric oxyhydroxide) that potentially affect gut microbiota were not evaluated in this study [24,53,54]. Fourth, the study was performed on Asian HD patients whose diet may be different from other populations. Last, to avoid the mixed effect of different phosphate binders on gut microbiota, participants ≥ two phosphate binders exposure were excluded. Furthermore, participants with ferric citrate or calcium carbonate treatment for more than two months can represent a single phosphate binder user in our study. Nevertheless, we only have a limited sample size with only eight ferric citrate users, so the results should be interpreted cautiously.

## 5. Conclusions

This study demonstrated that the diversity and composition of the gut microbiota were different in ferric citrate and calcium carbonate phosphate binders, confirming that different phosphate binders have additional effects, which may be mediated by the selective modulation of the microbiota. Further investigations are warranted for the cause–effect role of ferric citrate phosphate binder treatment on gut microbiota and subsequent manipulation of different phosphate binders to improve abnormal gut microbiota, thereby present promising targets for future studies.

## Figures and Tables

**Table 1:** Baseline characteristics of hemodialysis patients with different phosphate binders used.

Baseline Characteristics	Ferric Citrate Phosphate Binder (*n* = 8)	Calcium Carbonate Phosphate Binder (*n* = 46)	*p-*value
Age (years)	55.8 ± 11.8	61.5 ± 10.9	0.178
Male	5 (62.5%)	25 (54.3%)	0.668
Dialysis vintage (months)	91.5 ± 80.45	84.11 ± 69.53	0.787
Arteriovenous fistula	7 (87.5%)	44 (95.7%)	0.353
Cause of ESRD			
Hypertension	0 (0.0%)	10 (21.7%)	0.144
Diabetes mellitus	3 (37.5%)	12 (26.1%)	0.506
Glomerulonephritis	1 (12.5%)	14 (30.4%)	0.296
Others *	4 (50.0%)	10 (21.7%)	0.092
Comorbidities			
Diabetes mellitus	3 (37.5%)	15 (32.6%)	0.786
Hypertension	7 (87.5%)	38 (82.6%)	0.732
Dyslipidemia	2 (25.0%)	14 (30.4%)	0.756
Coronary artery disease	1 (12.5%)	3 (6.5%)	0.551
Cerebrovascular disease	1 (12.5%)	10 (21.7%)	0.549
Parathyroidectomy	2 (25.0%)	8 (17.4%)	0.609
Medications			
Antiplatelet	3 (37.5%)	13 (28.3%)	0.597
Antihypertensive drugs	5 (62.5%)	25 (54.3%)	0.668
Diabetes treatment medications	1 (12.5%)	13 (28.3%)	0.348
Clinical laboratory data			
Hemoglobin (g/dL)	10.83 ± 1.38	10.89 ± 0.99	0.870
Albumin (g/dl)	3.87 ± 0.38	3.92 ± 0.34	0.672
Alkaline phosphatase (IU/L)	66.96 ± 15.35	76.25 ± 26.98	0.350
Ion calcium (mg/dL)	61.5 ± 10.94	71.06 ± 28.64	0.359
Phosphate (mg/dL)	5.13 ± 0.75	6.88 ± 8.68	0.575
Parathyroid hormone (pg/mL)	403.15 ± 206.76	283.16 ± 277.95	0.250
Aluminum (ng/mL)	13.25 ± 5.44	13.28 ± 6.47	0.991
Magnesium (mg/dL)	2.66 ± 0.42	8.44 ± 21.54	0.455
Single pool Kt/V	1.44 ± 0.15	1.53 ± 0.24	0.298
Dietary intake (serving/day)			
Meat	0.7 ± 0.38	0.8 ± 0.54	0.760
Vegetable	1.3 ± 0.52	1.3 ± 0.60	0.852
Fruit	0.9 ± 0.62	1.1 ± 0.89	0.903
Bristol stool scale	4.5 ± 1.60	3.74 ± 1.82	0.273

* Other causes of end-stage renal disease include polycystic kidney disease, tumor, systemic lupus erythematosus, gout, and interstitial nephritis. Abbreviation: ESRD, end-stage renal disease.

## Supplementary Materials

The following are available online at https://www.mdpi.com/2076-2607/8/12/2040/s1, Figure S1: Enrollment of study participants, Figure S2: Rarefaction curves show the number of sequence reads and their corresponding number of OTUs, Figure S3: Core microbiota associated with ferric citrate phosphate binder used in hemodialysis patients. The name of the microbiome was summarized in Table S1, Figure S4: Core microbiota associated with calcium carbonate used in hemodialysis patients. The name of the microbiome was summarized in Table S2, Figure S5: The relative abundance of the specific genus (A), family (B), and order (C) differentially enriched in the clinical settings after linear discriminant analysis, Figure S6: Functional classification of the predicted metagenome content of the microbiota of two different phosphate binders using KO modules. The relative abundances of modules were compared between hemodialysis patients with ferric citrate phosphate binder used or calcium carbonate phosphate binder used. Significance was considered for *p* < 0.05, Table S1: Summary table of core microbiota associated with ferric citrate phosphate binder used in hemodialysis patients, Table S2: Summary table of core microbiota associated with calcium carbonate phosphate binder used in hemodialysis patients.
